# Factor analysis of defecation status in the general elderly population: the Yamagata Cohort Study

**DOI:** 10.1265/ehpm.25-00309

**Published:** 2026-03-04

**Authors:** Hitomi Kataoka, Tsuneo Konta, Yu Sasaki, Yoshiyuki Ueno

**Affiliations:** 1 Department of Fundamental Nursing, Yamagata University Graduate School of Medicine, 2-2-2 Iida-Nishi, Yamagata 990-9585, Japan; 2 Department of Public Health and Hygiene, Yamagata University Graduate School of Medical Science, 2-2-2 Iida-Nishi, Yamagata 990-9585, Japan; 3 Department of Gastroenterology, Yamagata University Faculty of Medicine, 2-2-2 Iida-Nishi, Yamagata 990-9585, Japan

**Keywords:** Constipation, Cohort, Quality of life, Older Japanese adults, Cross-sectional study

## Abstract

**Background:**

The prevalence of constipation is increasing with the aging of the population, significantly impacting the quality of life in older adults. However, a comprehensive analysis of the physical, psychological, and social correlates of constipation in Japan remains limited. This study aimed to examine factors associated with defecation status in community-dwelling older adults in Japan.

**Methods:**

We conducted a cross-sectional analysis of 8,009 community-dwelling residents aged 65 years and older who completed the baseline health and lifestyle questionnaire as part of the Yamagata Cohort Study. Constipation was defined as fewer than three bowel movements per week. Physical, psychological, and social factors were assessed using self-administered questionnaires. Associations with constipation were examined using chi-square tests and multivariable logistic regression, including sex-stratified models.

**Results:**

Constipation was observed in 407 participants (5.1%). In the multivariable analysis, it was significantly associated with being female (odds ratio [OR] = 1.26, 95% confidence interval [CI]: 1.02–1.56; reference: male), having a body mass index (BMI) ≥25 kg/m^2^ (OR = 0.74, 95% CI: 0.58–0.96: reference: <25 kg/m^2^), feeling unhappy (OR = 1.94, 95% CI: 1.16–3.22; reference: happy), experiencing residual stool sensation (OR = 2.77, 95% CI: 2.11–3.64; reference: no sensation), having moderate overactive bladder (OAB) symptoms (OR = 1.42, 95% CI: 1.13–1.79; reference: no OAB symptoms), and perceived living situation on current income as difficult (OR = 1.70, 95% CI: 1.36–2.12; reference: comfortable). Sex-stratified analyses revealed partially different patterns of associated factors between men and women.

**Conclusion:**

Constipation in community-dwelling older adults in Japan was associated with multiple physical, psychological, and social factors, with sex-specific differences. Given the cross-sectional design, causality cannot be inferred. These findings suggest that constipation in older adults may be associated with a complex interplay of physical, psychological, and social factors, highlighting the need for comprehensive and integrated approaches in community settings. Integrated geriatric care that addresses these dimensions may be effective in preventing and managing constipation in this population.

**Supplementary information:**

The online version contains supplementary material available at https://doi.org/10.1265/ehpm.25-00309.

## Background

The prevalence of constipation in Japan is increasing with the aging of the population. According to the 2022 National Population Survey, 7.12% of individuals aged ≥65 years experience constipation [1]. The etiology of constipation may involve obstruction or stricture due to malignancy or other causes, bowel dysfunction or impaired defecation, or is unknown. Several contributing factors have been identified, including an irregular or unbalanced diet, sedentary lifestyle, psychological stress, and medication use. Constipation is more common in older adults because of age-related decrease in abdominal muscle strength, inadequate dietary intake, insufficient fluid consumption, and decreased physical activity [2].

The Japanese Guidelines for the Diagnosis and Treatment of Constipation Disorders: Chronic Constipation 2023 [3] defines constipation as a state of fecal impaction or hard stools due to retention of feces in the colon, decreased frequency of bowel movements, excessive irritation due to inability to defecate comfortably, a feeling of residual fecal impaction, rectal and anal obstruction, and a feeling of difficulty defecating. Constipation is classified into two types: (1) decreased defecation frequency where stool remains in the colon, and (2) difficult defecation, where stool passage in the rectum is impaired. A key diagnostic criterion for chronic constipation is spontaneous defecation of fewer than three times per week.

Constipation significantly impacts quality of life. A systematic review assessing quality of life in patients with chronic constipation using the Short Form36 and 12 health surveys reported that constipation has a greater impact on mental health than on physical health at a magnitude comparable to that of allergies, musculoskeletal disorders, and inflammatory bowel disease [4]. In a Swedish geriatric care facility, the prevalence of constipation was 67%, with significantly higher rates among individuals with cognitive impairment, physical disability, use of physical restraint, speech impairment, nutritional disorders, and polypharmacy [5]. The prevalence of constipation among the elderly people in China was 17.6%, with a higher incidence in women and those with shorter nighttime sleep duration [6]. In Japanese women, functional constipation has been linked to moderate-to-severe overactive bladder and urge incontinence [7, 8]. Despite the known impact of constipation on various aspects of quality of life, the physical, psychological, and social factors associated with chronic constipation in elderly Japanese individuals remain underexplored. In addition, lifestyle characteristics in Japan, such as vegetable-rich diets and rural living patterns, may promote more regular bowel movements, which could partly explain the relatively low prevalence of constipation observed in prior community-based studies.

The Yamagata Cohort Study questionnaire collected data on physical, psychological, and social indicators in a large, community-based sample of older Japanese adults. In this analysis, constipation was defined as fewer than three bowel movements per week, based on the questionnaire item available in the cohort study. Although this frequency-based definition does not encompass the full symptom dimensions outline in the Rome IV [9] criteria (e.g., straining, stool consistency, incomplete evacuation), it represents a widely used and guideline-consistent indicator of chronic constipation. The purpose of this study is to investigate the factors associated with constipation, defined pragmatically as fewer than three bowel movements per week, and to evaluate potential sex-specific differences. By addressing multidimensional correlates, our findings add to the understanding of constipation beyond clinical and biological determinants, highlighting psychosocial influences relevant for preventive strategies and geriatric care in community settings.

## Methods

### Study design and participants

Of the 12,208 community-dwelling residents aged 65 years and older who voluntarily participated in the baseline questionnaire survey of the 2021–2022 Yamagata Cohort Study, 8,009 respondents who provided complete data on defecation status, daily living, and health-related indicators were included in the present cross-sectional analysis. This study was approved by the Ethical Review Committee of Yamagata University School of Medicine (Approval No. 2022-274), and all participants provided written informed consent.

### Measurements

Data on physical (age, sex, height, weight, walking problems, weight loss in the past 6 months, abdominal fullness, frequency of bowel movements, residual stool sensation, urinary status), psychological (feeling of happiness), and social (sleep duration, perceived living situation on current income) factors were obtained from the Health and Lifestyle Questionnaire.

Variables were categorized as follows:

• Age (65–69, 70–74, 75–79, >80 years)
• Body mass index (BMI), calculated from self-reported height and weight: (<25 kg/m^2^, ≥25 kg/m^2^)
• Walking problems (yes, no)
• Feeling of happiness (happy, neutral, unhappy)
• Weight loss in the past 6 months (yes, no)
• Sleep duration (≤6 hours, ≥7 hours)
• Abdominal fullness (yes, no)
• Residual stool sensation (yes, no)
• Overactive bladder (OAB) according to the Overactive Bladder Syndrome Score (OABSS)
  OABSS is based on four items (daytime frequency, nocturia, urgency, and urge incontinence). OAB is defined as a total score of ≥3, with an urgency score (Q3) of ≥2. Severity is categorized as follows: 0 or not meeting the criteria was categorized as having no symptoms; a total score 3–5 was categorized as mild; a total score 6–11 was categorized as moderate; and a total score 12–15 was categorized as severe, according to established criteria [10].
• Perceived living situation on current income (comfortable, neutral, difficult)

The exact wording and response scale for each item are provided in Supplementary Table S1. Age and BMI were calculated from self-reported values and not included in the table.

### Constipation definition

Defecation frequency was assessed with a single questionnaire item and categorized into two groups: fewer than three bowel movement per week versus three or more bowel movement per week. Participants with a frequency of fewer than three bowel movements per week were classified as having constipation. Although the questionnaire included several items related to bowel symptoms (e.g., sensation of complete evacuation, perceived constipation, stress-related constipation, urgency, stool consistency), these items referred only to the past week and did not fully align with the Rome IV [9] diagnostic criteria for chronic constipation. According to the Rome IV [9] framework, the diagnostic criteria require at least two symptoms persisting for the past three months and that symptom onset occurred at least six months prior to diagnosis. Therefore, in this study, constipation was pragmatically defined based on bowel movement frequency alone (fewer than three bowel movement per week), which aligns with both the Japanese Clinical Practice Guidelines for Chronic Constipation (2023) [3] and one key element of the Rome IV [9] criteria. The potential implication of this limitation is addressed in the Discussion section.

### Statistical analysis

Categorical variables were compared using the chi-squared test. Multivariable logistic regression analysis was performed to identify factors associated with constipation. Full coding and reference categories for all predictors are provided in Supplementary Table S1. Missing data were handled using complete case analysis. Variable selection was based on previous studies and univariate screening.

Model diagnostics were conducted to assess multicollinearity and model fit. Additionally, sex-specific differences, interaction terms (sex * age and sex * OAB) were tested in a combined model.

All statistical analyses were performed using with JMP Software student edition 18 for Windows (SAS Institute Japan Ltd., Tokyo, Japan). A two-side p-value <0.05 was considered statistically significant.

## Results

### Characteristics of study participants

Among the total study population, most participants were aged 70–74 years (n = 2,772; 34.6%), followed by 75–79 years (n = 2,332; 29.1%), ≥80 years (n = 1,517; 19.0%), and 65–69 years (n = 1,388; 17.3%). The cohort included 3,467 (43.3%) male and 4,542 (56.7%) female individuals. Constipation was observed in 407 (5.1%) participants. Table 1 presents the demographic characteristic of the participants (Table 1).

**Table 1:** Baseline characteristics of study participants

**Category**	**Total**	**<3 bowel movements per week** **n = 407 (5.1%)**	**≥3 bowel movements per week** **n = 7602 (94.9%)**	**P value**
	**n (%)**	**n (%)**	**n (%)**	
Age				
65–69	1388 (17.3)	75 (18.4)	1313 (17.3)	0.05
70–74	2772 (34.6)	137 (33.7)	2635 (34.7)	
75–79	2332 (29.1)	100 (24.6)	2232 (29.3)	
≥80	1517 (19.0)	95 (23.3)	1422 (18.7)	
Sex				
Male	3467 (43.3)	161 (39.6)	3306 (43.5)	0.119
Female	4542 (56.7)	246 (60.4)	4296 (56.5)	
BMI				
<25	2187 (27.3)	324 (79.6)	1863 (24.5)	0.059
≥25	5822 (72.7)	83 (20.4)	5739 (75.5)	
Walking problems				
No	5666 (70.7)	250 (61.4)	5416 (71.2)	<0.001
Yes	2343 (29.3)	157 (38.6)	2186 (28.8)	
Feeling of happiness				
Happy	6933 (86.6)	322 (79.1)	6611 (87.0)	<0.001
Neutral	901 (11.2)	60 (14.7)	841 (11.0)	
Unhappy	175 (2.2)	25 (6.2)	150 (2.0)	
Weight loss in the past 6 months				
No	1056 (13.2)	72 (17.7)	984 (12.9)	0.006
Yes	6953 (86.8)	335 (82.3)	6618 (87.1)	
Sleep duration				
≤6 hours	1546 (19.3)	81 (19.9)	1465 (19.3)	0.754
≥7 hours	6463 (80.7)	326 (80.1)	6137 (80.7)	
Abdominal fullness				
No	5553 (69.3)	252 (61.9)	5301 (69.7)	0.001
Yes	2456 (30.7)	155 (38.1)	2301 (30.3)	
Residual stool sensation				
No	3026 (37.8)	70 (17.2)	2956 (38.9)	
Yes	4983 (62.2)	337 (82.8)	4646 (61.1)	
Overactive bladder syndrome score				
No symptoms	6305 (78.7)	283 (69.5)	6022 (79.2)	<0.001
Moderate	1624 (20.3)	119 (29.2)	1505 (19.8)	
Severe	80 (1.0)	5 (1.3)	75 (1.0)	
Perceived living situation on current income				
Comfortable	829 (10.3)	36 (8.8)	793 (10.4)	<0.001
Neutral	4882 (61.0)	197 (48.4)	4685 (61.6)	
Difficult	2298 (28.7)	174 (42.8)	2124 (28.0)	

Note: Mild category in overactive bladder syndrome score was not observed in either group.

### Factors associated with constipation

We conducted logistic regression analyses to identify factors associated with constipation. In the univariable analysis, constipation was significantly associated with walking problems (odds ratio [OR] = 1.56, 95% confidence interval [CI]: 1.27–1.91), weight loss in the past 6 months (OR = 0.69, 95% CI: 0.53–0.90), experiencing abdominal fullness (OR = 1.42, 95% CI: 1.15–1.74), experiencing residual stool sensation (OR = 3.06, 95% CI: 2.36–3.98), and having moderate OAB symptoms (OR = 1.68, 95% CI: 1.35–2.10) compared to those with no OAB symptoms.

Regarding psychological and social factors, participants who reported feeling neutral had lower odds of constipation (OR = 0.68, 95% CI: 0.51–0.91) compared to those who reported feeling happy, while those who reported feeling unhappy had higher odds (OR = 2.34, 95% CI: 1.42–3.90). Similarly, participants who reported perceived living situation on current income as difficult had increased odds of constipation compared to those who reported comfortable (OR = 1.94, 95% CI: 1.58–2.40). The results are presented in Table 2.

**Table 2:** Factors associated with fewer than three bowel movements per week

		**Univariable analysis**		**Multivariable analysis**	
		**OR (95%CI)**	**P value**	**OR (95%CI)**	**P value**
Age	65–69	Reference			
	70–74	0.91 (0.68–1.21)	0.524	0.94 (0.70–1.26)	0.661
	75–79	0.78 (0.58–1.06)	0.121	0.79 (0.57–1.08)	0.138
	≥80	1.17 (0.86–1.60)	0.325	1.17 (0.84–1.62)	0.363
Sex	Male	Reference			
	Female	1.18 (0.96–1.44)	0.12	1.26 (1.02–1.56)	0.031
BMI	<25	Reference			
	≥25	0.80 (0.61–1.00)	0.06	0.74 (0.58–0.96)	0.022
Walking problems					
	Yes	1.56 (1.27–1.91)	<0.001	1.23 (0.98–1.54)	0.07
Feeling of happiness					
	Happy	Reference			
	Neutral	0.68 (0.51–0.91)	0.009	0.90 (0.66–1.21)	0.467
	Unhappy	2.34 (1.42–3.90)	0.001	1.94 (1.16–3.22)	0.001
Weight loss in the past 6 months					
	Yes	0.69 (0.53–0.90)	0.006	0.80 (0.61–1.05)	0.114
Sleep duration					
	≤6 hours	Reference			
	≥7 hours	0.96 (0.75–1.23)	0.75	1.09 (0.85–1.42)	0.483
Abdominal fullness					
	Yes	1.42 (1.15–1.74)	0.001	0.96 (0.77–1.20)	0.757
Residual stool sensation					
	Yes	3.06 (2.36–3.98)	<0.001	2.77 (2.11–3.64)	<0.001
Overactive bladder syndrome score					
	No symptoms	Reference			
	Moderate	1.68 (1.35–2.10)	<0.001	1.42 (1.13–1.79)	0.003
	Severe	1.42 (0.57–3.56)	0.453	0.95 (0.37–2.42)	0.92
Perceived living situation on current income					
	Comfortable	Reference			
	Neutral	1.08 (0.75–1.55)	0.679	1.12 (0.77–1.61)	0.558
	Difficult	1.94 (1.58–2.40)	<0.001	1.70 (1.36–2.12)	<0.001

OR: odds ratio, CI: confidence interval

In the multivariable analysis, constipation was significantly associated with being female (OR = 1.26, 95% CI: 1.02–1.56), having a BMI ≥25, (OR = 0.74, 95% CI: 0.58–0.96), feeling unhappy (OR = 1.94, 95% CI: 1.16–3.22) compared to felling happy, experiencing residual stool sensation (OR = 2.77, 95% CI: 2.11–3.64), having moderate OAB symptoms (OR = 1.42, 95% CI: 1.13–1.79) compared to no OAB symptoms, and perceived living situation on current income as difficult (OR = 1.70, 95% CI: 1.36–2.12) compared to those who reported comfortable (Table 2).

Model diagnostics confirmed the adequacy of the multivariable logistic regression analysis. The number of events per predictor variable was 57.1, indicating sufficient model power. No multicollinearity was detected, with all pairwise correlation coefficients below 0.2. The model showed discriminative ability with an are under the ROC curve (AUC = 0.683). Although the Hosmer-Lemeshow test could not be performed using JMP student edition 18 and visual inspection of the ROC curve suggested no substantial lack of fit (see Supplementary Table S2). Additionally, to explore sex-related differences in associations, we tested formal interaction terms for Sex * Age and Sex * OAB in a combined model. None of the interaction terms were statistically significant (all p-values >0.05; for example, sex*age p = 0.100). This suggests that the sex-stratified differences observed in Tables 2 and 3 are more likely to reflect differences in effect sizes than to indicate formal effect modification.

**Table 3:** Factors associated with fewer than three bowel movements per week by gender

		**Male**		**Female**	
		**OR (95%CI)**	**P value**	**OR (95%CI)**	**P value**
Age	65–69	Reference		Reference	
	70–74	1.56 (0.84–2.89)	0.16	0.80 (0.57–1.13)	0.214
	75–79	1.34 (0.72–2.5)	0.36	0.64 (0.43–0.95)	0.027
	≥80	1.58 (0.84–3.0)	0.16	1.16 (0.77–1.75)	0.473
BMI	<25	Reference			
	≥25	0.73 (0.50–1.07)	0.105	0.77 (0.54–1.08)	0.13
Walking problems					
	Yes	1.61 (1.14–2.28)	0.007	1.03 (0.77–1.38)	0.843
Feeling of happiness					
	Happy	Reference		Reference	
	Neutral	1.22 (0.76–1.98)	0.408	0.71 (0.49–1.05)	0.091
	Unhappy	3.34 (1.62–6.89)	0.001	1.19 (0.56–2.52)	0.656
Weight loss in the last 6 months					
	Yes	0.77 (0.51–1.16)	0.204	0.83 (0.58–1.20)	0.323
Sleep duration					
	≤6 hours	Reference		Reference	
	≥7 hours	1.36 (0.85–2.17)	0.194	1.00 (0.73–1.36)	0.998
Abdominal fullness					
	Yes	1.13 (0.80–1.60)	0.486	0.85 (0.64–1.13)	0.272
Residual stool sensation					
	Yes	2.29 (1.48–3.56)	<0.001	3.12 (2.20–4.43)	<0.001
Overactive bladder syndrome score					
	No symptoms	Reference		Reference	
	Moderate	1.62 (1.15–2.30)	0.006	1.26 (0.92–1.74)	0.153
	Severe	1.29 (0.44–3.78)	0.64	0.54 (0.07–4.09)	0.55
Perceived living situation on current income					
	Comfortable	Reference		Reference	
	Neutral	1.35 (0.74–2.44)	0.326	0.99 (0.62–1.58)	0.961
	Difficult	2.25 (1.58–3.21)	<0.001	1.41 (1.06–1.88)	0.019

Multivariable analysis

OR: odds ratio, CI: confidence interval

### Factors associated with frequency of bowel movement by gender

We further conducted multivariable logistic regression analyses to determined sex-based differences. Among men, constipation was significantly associated with walking problems (OR = 1.61, 95% CI: 1.14–2.28), feeling unhappy (OR = 3.34, 95% CI: 1.62–6.89) compared to feeling happy, experiencing residual stool sensation (OR = 2.29, 95% CI: 1.48–3.56), having moderate OAB symptoms (OR = 1.62, 95% CI: 1.15–2.30), and perceived living situation on current income as difficult (OR = 2.25, 95% CI: 1.58–3.21).

Among women, significant associations were observed with being aged 75–79 years (OR = 0.64, 95% CI: 0.43–0.95) compared to those aged 65–69, experiencing residual stool sensation (OR = 3.12, 95% CI: 2.20–4.43), and perceived living situation on current income as difficult (OR = 1.41, 95%CI: 1.06–1.88) (Table 3).

## Discussion

Constipation, defined as a defecation frequency of fewer than three bowel movements per week, was observed in 5.1% older Japanese adults. Several multidimensional factors-including lower BMI, unhappiness, residual stool sensation, moderate OAB, and perceived living situation on current income as difficult were significantly associated with constipation, with partially different patterns by sex.

Previous studies have reported a wide variation in chronic constipation prevalence, ranging from 2% to 27%, which is caused by differences in study populations, definitions, and assessment methods [11]. In a cross-sectional survey of individuals aged 60–93 years, Salari et al. [12] reported a prevalence of 18.9% worldwide and 13.6% in Asia. Similarly, Du et al. [6] reported a prevalence of 17.6% among adults aged ≥65 years in China. In Japan, a 2022 survey conducted by the Ministry of Health, Labor and Welfare reported a prevalence of 7.12% among adults aged ≥65 years [1]. In this study, the prevalence of constipation was 5.1%, which is relatively lower than many international reports. This discrepancy may reflect differences in population characteristics, study design, and diagnostic criteria. Our reliance on a frequent-based definition likely underestimated the prevalence compared to Rome IV [9] criteria, which incorporate additional symptom dimensions. Nevertheless, frequency-based definitions are widely used in epidemiological research and are consistent with current guideline recommendation. Previous studies have reported variability in constipation prevalence across populations. For instance, one study suggested that vegetarians and vegans defecate more frequently than meat-eaters [13], whereas another found that prevalence rates vary based on factors such as sex, age, geographic region, survey methodology, and cultural characteristics [14]. Because the current study population comprised older adults living in rural areas, who may be more physically active and consume more vegetables, these lifestyle factors might have contributed to the relatively low constipation rate observed in our cohort and may limit the generalizability of findings to other populations.

Constipation affects quality of life [4] and frequently coexists with depression and anxiety [15]. In this study, feeling of unhappiness were significantly associated with constipation, particularly in men. Furthermore, perceived living situation on current income as difficult was linked to constipation in both men and women, signifying that psychological and social factors affect bowel function. These psychological and social correlates, including unhappiness and financial difficulty, were observed and may suggest the involvement of the brain-gut axis and psychosocial stressors in bowel function. Psychological factors may contribute to constipation through the brain-gut axis, which involves neurological, neuro-immunological, and neuroendocrine pathways [16]. The corticotropin-releasing factor pathway, which is implicated in depression, can disrupt the brain-gut axis interactions and may increase the risk of constipation [17]. Furthermore, serotonin, a neurotransmitter that regulates mood, sleep, appetite, physical activity, and body temperature, may possibly modulate the relationship between physical symptoms and constipation [18]. Further studies are needed to elucidate the mechanisms by which psychological and social factors influence bowel function.

Constipation has been identified as a risk for stress urinary incontinence and OAB in Japanese [7, 8] and Chinese women [19]. The present study confirmed a similar association based on more objective indicators. Regarding the link between defecation status and urinary flow, a previous study reported that colonic distention induces increased bladder contractility, distention, and vascular reflex [20]. Furthermore, chronic colonic distention caused by constipation and exacerbate irritable bladder symptoms [7]. Stool accumulation in the rectum increase abdominal pressure and rectal dilation, potentially causing damage to the pelvic floor [8]. The association between constipation and OAB highlights the need for comprehensive health counselling addressing both defecation and urination functions. In the present study, a significant association between constipation and moderate OAB was observed only in men. This sex-specific finding aligns with previous studies reporting a link between OAB and prostatic hyperplasia in older men [17]. While our study did not directly assess prostate-related factors, these results suggest that lower urinary tract conditions such as benign prostatic hyperplasia may contribute to the observed association. Further research incorporating urological assessments is necessary to explore this potential mechanism. To address sex-specific differences, we additionally tested interaction terms (sex * age and sex * OAB) in a combined model. None of the interaction and OAB or age did not differ significantly by sex. However, we acknowledge that some interaction terms showed borderline p-values (e.g., sex* age p = 0.100), which may reflect potential trends worth further exploration in large samples. These results indicate that sex-stratified findings observed in our study, such as the association between moderate OAB and constipation in men or the age-related effect in women, may reflect differences in effect sizes rather than true statistical interaction. Future longitudinal analysis will be helpful in disentangling these patterns.

Residual stool sensation was associated with constipation in both men and women. According to the Evidence-Based Clinical Practice Guidelines for Chronic Constipation 2023 [3], recto-anal dysfunction such as hyposensitivity of the rectum and defecation coordination disorder (dyssynergic defecation), is involved in chronic constipation, contributing to dyspareunia and residual stool sensation. The findings of this study suggest that residual stool sensation may be associated with dyssynergic defecation in the older adults.

A study on the prevalence and risk factors for functional constipation in Japanese individuals reported that BMI was lower in those with functional constipation group [21]. Furthermore, a positive association between BMI and increased defecation frequency has also been reported [13]. The findings of the present study on the association between lower BMI and constipation aligns with those of the previous reports.

The strengths of this study include its large sample size and the comprehensive assessment of physical, psychological, and social factors associated with constipation. However, this study has some limitations. First, cross-sectional design precludes causal inference and raises the possibility of reverse causation, such as constipation influencing psychological well-being or daily activity. Moreover, it is possible that the direction and strength of these mechanisms, such as reverse causation and unmeasured confounding, may differ by sex. For example, anxiety or urinary symptoms may influence bowel habits differently in men and women. These considerations further support the need for future sex-specific longitudinal studies to clarify causal pathways. Second, potential confounders-including comorbidities, diet, fiber intake, hydration, and medication use-were not available in this dataset. Third, the frequency-based definition used here does not fully capture Rome IV [9] symptom dimensions. This pragmatic definition did not incorporate other chronic symptom dimensions (e.g., straining, hard stool, incomplete defecation), which could have led to underestimation of prevalence or misclassification. Likewise, key psychosocial variables such as happiness and perceived living situation on current income as difficult were assessed via single item self-reports, without standardized validation. Finally, participants were volunteers from predominantly rural communities. This rural volunteer sample may limit generalizability to urban, institutionalized, or non-Japanese populations, and may partly account for the relatively low prevalence of constipation observed in our study.

## Conclusions

In this cross-sectional study, constipation in community-dwelling older adults in Japan was associated with multiple physical, psychological, and social factors, with sex-specific differences. Key correlates included OAB, lower BMI, residual stool sensation, unhappiness, and financial difficulty, with partially different patterns between men and women. These findings suggest the constipation in older adults may reflect multidimensional influences beyond biological determinants, highlighting the need for further research on comprehensive approaches in community settings.

## Abbreviations

BMI: Body mass index
OAB: Overactive bladder
OABSS: Overactive Bladder Syndrome Score
OR: odds ratio, CI: confidence interval

## Supplementary information

**Additional file 1:** **Supplementary Table S1** Questionnaire items and response options used in the analysis. **Supplementary Table S2** Model diagnostics for multivariable logistic regression analysis.

## Declarations

### Availability of data and materials

The dataset used in this study is not publicly available due to ethical restrictions, but it can be obtained from the corresponding author upon reasonable request.
