# Supplementary material for: Factor analysis of defecation status in the general elderly population: the Yamagata Cohort Study
Source: Environ Health Prev Med. 2026 Mar 4;31:16. doi: 10.1265/ehpm.25-00309 (PMC12981972; doi:10.1265/ehpm.25-00309)
Supplement: Supplementary file 1 — Additional file 1: Supplementary Table S1 Questionnaire items and response options used in the analysis. Supplementary Table S2 Model diagnostics for multivariable logistic regression analysis. [file ehpm-31-016-s001.docx]

| Supplementary Table S1　Questionnaire items and response options used in the analysis | | | |
| --- | --- | --- | --- |
| Category | Questionnaire item | Response option | Note |
| Walking problems | Do you have any problems walking around? | (1) No problems at all  (2) Slight problems  (3) Moderate problems  (4) Severe problems  (5) Unable to walk | (1) No  (2)-(5) Yes  Self-reported |
| Feeling of happiness | How happy do you feel about your life overall? | (1) Very happy  (2) Fairly happy  (3) Neither  (4) Not very happy  (5) Not happy at all | (1)-(2) Happy  (3) Neutral  (4)-(5) Unhappy  Self-reported |
| Weight loss in the past 6 months | Have you lost 2-3kg or more in the past 6 months? | Yes  No | Self-reported |
| Sleep duration | How many hours do you usually sleep per night? | (1) ≤ 6 hours  (2) 7 hours  (3) 8 hours  (4) 9 hours  (5) 10 hours  (6) ≥ 11 hours | Categorized  ≤ 6 hours vs  ≥ 7 hours  Self-reported |
| Abdominal fullness | Have you been troubled by a feeling of abdominal fullness or bloating? | (0) Not at all  (1) Hardly  (2) A little  (3) Somewhat  (4) Quite a bit  (5) Extremely | (0) No  (1)-(5) Yes  Self-reported |
| Residual stool sensation | Have you been troubled by a feeling of incomplete bowel evacuation (residual stool sensation)? | (0) Not at all  (1) Hardly  (2) A little  (3) Somewhat  (4) Quite a bit  (5) Extremely | (0) No  (1)-(5) Yes  Self-reported |
| Overactive bladder syndrome score | Assessed using the Overactive Bladder Symptom Score (OABSS), based on four items: daytime frequency, nocturia, urgency, and urgency incontinence | Total score  0 or not meeting criteria:  No symptoms  3-5: Mild  6-11: Moderate  12-15: Severe | Defined as total score ≥3 and ≥2 (Q3: urgency).  Categorized per OABSS guidelines (Homma et al., 2006)^10)^  Validated scale |
| Perceived living situation on current income | How would you describe current living situation based on your income? | (1) Very comfortable  (2) Comfortable  (3) Average  (4) Somewhat difficult  (5) Very difficult | (1)-(2) Comfortable  (3) Neutral  (4)-(5) Difficult  Self-reported |

Age and BMI were calculated from self-reported values.

| Supplementary Table S2　 Model diagnostics for multivariable logistic regression analysis | |
| --- | --- |
| items | Details |
| Events per variable (EPV) | 800 events / 14 predictors = 57.1 |
| Multicollinearity | All pairwise correlation coefficients <0.2 |
| Model discrimination (AUC) | AUC = 0.683 (from ROC curve) |
| Model calibration | Hosmer-Lemeshow test not available in JPM student edition;  Visual inspection of ROC curve showed no sign of poor fit |
